# Could full thickness skin grafts in an onlay position be the new gold standard for incisional hernia repair? Author’s reply

**DOI:** 10.1007/s10029-022-02589-8

**Published:** 2022-03-17

**Authors:** V. Holmdahl, B. Stark, L. Clay, U. Gunnarsson, K. Strigård

**Affiliations:** 1 Department of Surgical and Perioperative Sciences, Surgery, Sunderby Research Unit, Umeå University, Daniel Naezéns väg, 90185 Umeå, Sweden; 2 Department of Plastic and Reconstructive Surgery, Karolinska University Hospital, MK1 Karolinska Institute, Stockholm, Sweden; 3 Department of Clinical Science and Education Södersjukhuset, Karolinska Institute, Stockholm, Sweden; 4 Department of Surgery, Södersjukhuset, Stockholm, Sweden; 5 Department of Surgical and Perioperative Sciences, Surgery, Umeå University, Umeå, Sweden

We thank Prof Berrevoet for his interest in our trial and his comments on the potential use of full-thickness skin grafting in hernia repair [1, 2].

We agree that sample size of the trial potentially decreases the statistical power of outcomes other than the main endpoint, thus increasing the risk of type 2 error. The main outcome of our trial was surgical complication at the 3-month follow-up. The lack of differences in other outcomes must therefore be interpreted with caution, and we addressed this issue in both the one-year and long-term follow-up publications. However, considering that the present trial included only the subgroup of incisional hernias deemed “giant”, the study population can be considered large, even for a tertiary referral center. This is the main reason for the relatively long inclusion time.

Regarding the heterogenicity of the techniques used, the state of the art at the start of the trial must be considered. Inclusion began December 2009, and investigations on the behavior of full-thickness skin in the various positions of the abdominal wall were performed several years later [3]. This limited the full-thickness skin graft positioning in our trial to the onlay position as used in the proof-of-concept study, when in fact the sublay position may have been a better alternative [4]. Prof. Berrevoet mentions transversus abdominis release, which is a technique that is gaining in popularity, but this was introduced 2012 and could not have been used in the present trial [5]. However, new approaches and increasing knowledge in the treatment of incisional hernia also opens for new applications of full-thickness skin grafting. A final aspect is the increasing hesitancy amongst certain patient groups regarding implantation of synthetic materials. This highlights the need for development of alternative strategies such as full-thickness skin grafting.

We also agree with Dr. Berrevoet, that trial results should not be spread out over an unnecessarily large number of publications, which may negatively affect readability and make it difficult to gain an overview of the outcome of the trial. We believe, however, that results of a trial should be published as soon as data are available so that as many patients benefit as soon as possible. This may result in several different publications from the same trial.

Generally, the main problem with hernia surgery research is more the lack of long-term follow-up publications than the fact that long-term data result in more than one publication.

Sincerely yours,

## References

[1] Holmdahl V, Stark B, Clay L, Gunnarsson U, Strigård K (2021). Long-term follow-up of full-thickness skin grafting in giant incisional hernia repair: a randomised controlled trial. Hernia.

[2] Berrevoet F (2022). Could full thickness skin grafts in an onlay position be the new gold standard for incisional hernia repair?. Hernia.

[3] Winsnes A, Gunnarsson U, Falk P, Stark B, Moskaug JØ, Strigård K (2018). Evaluating full-thickness skin grafts in intraperitoneal onlay mesh position versus onlay position in mice. J Surg Res.

[4] Strigård K, Stark B (2008). Repair of giant abdominal wall hernias with full-thickness skin transplants in high-risk patients. Eur J Plast Surg.

[5] Novitsky YW, Elliott HL, Orenstein SB, Rosen MJ (2012). Transversus abdominis muscle release: a novel approach to posterior component separation during complex abdominal wall reconstruction. Am J Surg.

